# Tissue Engineering of Cartilage Using a Random Positioning Machine

**DOI:** 10.3390/ijms21249596

**Published:** 2020-12-16

**Authors:** Markus Wehland, Paul Steinwerth, Ganna Aleshcheva, Jayashree Sahana, Ruth Hemmersbach, Ronald Lützenberg, Sascha Kopp, Manfred Infanger, Daniela Grimm

**Affiliations:** 1 Department of Microgravity and Translational Regenerative Medicine, Clinic for Plastic, Aesthetic and Hand Surgery, Otto von Guericke University, 39106 Magdeburg, Germany; paul.steinwerth@st.ovgu.de (P.S.); anna_alestheva@yahoo.de (G.A.); ronald.luetzenberg@med.ovgu.de (R.L.); sascha.kopp@med.ovgu.de (S.K.); manfred.infanger@med.ovgu.de (M.I.); daniela.grimm@med.ovgu.de (D.G.); 2 Department for Biomedicine, Aarhus University, 8000 Aarhus, Denmark; jaysaha@biomed.au.dk; 3 Gravitational Biology, Institute of Aerospace Medicine, German Aerospace Center, 51147 Cologne, Germany; ruth.hemmersbach@dlr.de

**Keywords:** tissue engineering, cartilage, spheroids, random positioning machine, scaffold-free

## Abstract

Articular cartilage is a skeletal tissue of avascular nature and limited self-repair capacity. Cartilage-degenerative diseases, such as osteoarthritis (OA), are difficult to treat and often necessitate joint replacement surgery. Cartilage is a tough but flexible material and relatively easy to damage. It is, therefore, of high interest to develop methods allowing chondrocytes to recolonize, to rebuild the cartilage and to restore joint functionality. Here we studied the in vitro production of cartilage-like tissue using human articular chondrocytes exposed to the Random Positioning Machine (RPM), a device to simulate certain aspects of microgravity on Earth. To screen early adoption reactions of chondrocytes exposed to the RPM, we performed quantitative real-time PCR analyses after 24 h on chondrocytes cultured in DMEM/F-12. A significant up-regulation in the gene expression of *IL6*, *RUNX2*, *RUNX3*, *SPP1*, *SOX6*, *SOX9*, and *MMP13* was detected, while the levels of *IL8*, *ACAN*, *PRG4*, *ITGB1*, *TGFB1*, *COL1A1*, *COL2A1*, *COL10A1*, *SOD3*, *SOX5*, *MMP1*, and *MMP2* mRNAs remained unchanged. The STRING (Search Tool for the Retrieval of Interacting Genes/Proteins) analysis demonstrated among others the importance of these differentially regulated genes for cartilage formation. Chondrocytes grown in DMEM/F-12 medium produced three-dimensional (3D) spheroids after five days without the addition of scaffolds. On day 28, the produced tissue constructs reached up to 2 mm in diameter. Using specific chondrocyte growth medium, similar results were achieved within 14 days. Spheroids from both types of culture media showed the typical cartilage morphology with aggrecan positivity. Intermediate filaments form clusters under RPM conditions as detected by vimentin staining after 7 d and 14 d. Larger meshes appear in the network in 28-day samples. Furthermore, they were able to form a confluent chondrocyte monolayer after being transferred back into cell culture flasks in 1 *g* conditions showing their suitability for transplantation into joints. Our results demonstrate that the cultivation medium has a direct influence on the velocity of tissue formation and tissue composition. The spheroids show properties that make them interesting candidates for cellular cartilage regeneration approaches in trauma and OA therapy.

## 1. Introduction

Adult articular cartilage tissue has only a very limited ability for self-regeneration owing to its low vascularization and cell density [1]. Therefore, even minor lesions may lead to progressive damage and degeneration (osteoarthritis (OA), cartilage trauma). The incidence of these disorders is steadily increasing. In the U.S. alone, more than 70 million patients suffer from articular cartilage injuries caused mainly by OA, generating an estimated economic burden of about $100 billion by 2020, as the elderly population continues to grow [2]. Three-dimensional (3D) cell culture techniques have therefore attracted much attention among clinicians interested in tissue engineering as a novel approach for cartilage repair.

Chondrocytes are able to produce, maintain, and remodel the cartilage extracellular matrix (ECM) in vitro [3]. However, chondrocyte expansion in monolayer cultures causes cell dedifferentiation and loss of their original phenotype after more than four rounds of subculture [4]. In most cartilage tissue engineering studies, chondrocytes from immature animals are used. These proliferate faster and have an increased chondrogenic potential compared to the cells from older animals or human donors, which are metabolically less active in vitro [5]. These limitations can partly be counteracted with altered culture conditions such as usage of rotating bioreactors [6], culture in serum-free media [7], growth under reduced oxygen tension [8], and the addition of growth factors [9]. However, unlike bone, cartilage regeneration using tissue engineering strategies has not yet been successful in precisely recreating cartilage which was both structurally and functionally equivalent to in vivo tissue [10].

Cell culture systems including spinner flasks, rotating bioreactors, and perfusion (flow-through) bioreactors have already been successfully applied for cartilage tissue engineering [11]. Bioreactors offer several important advantages over simple tissue-flask and Petri-dish culture systems. Bioreactors provide enhanced mass transfer by convective fluid flow, the ability to provide mechanical forces influencing tissue development, and a better control over culture conditions. In most of the cases, cartilage development is achieved in vitro by cultivating chondrocytes on biodegradable polymer scaffolds. These scaffolds provide an environment in which cells can proliferate, function, secrete proteins, and organize into a tissue that resembles natural cartilage in important respects [12]. The ideal scaffold should be biodegradable without exerting cytotoxic, tumorigenic, nephrotoxic or other undesirable effects; have a porosity that allows for diffusion of nutrients and waste products; support cell viability, proliferation, differentiation and ECM production; be able to attach to and integrate into the tissue at the defect site; and provide mechanical support [1,3]. This is very hard to achieve. Moreover, problems such as necrosis due to high-density cell culture and shear stress have not yet been solved using conventional stirred fermenters [4].

The number of cells that can be isolated from a clinical biopsy is not sufficient for cell-based therapies such as autologous cartilage implantation. Thus, an expansion of the chondrocytes is necessary. This is usually achieved in monolayer cell culture which potentially reduces their chondrogenic capacities [13]. This is especially problematic for scaffold-free methods that rely on the volume of cells to act as their own mechanical support [6].

The Random Positioning Machine (RPM) is a device that was developed to simulate certain aspects of microgravity (µ*g*) on Earth. It consists of two perpendicular and independently driven frames, which enable the rotation of a sample mounted to the center of the inner frame around all three axes in space, essentially providing a spherical rotational freedom [14]. This setup can be used as a 3D clinostat, driving both frames with constant directions and speeds, or as an RPM, randomly varying both speed and direction. For a sufficiently small rotated sample (such as human cells) the direction of the gravity vector will constantly change, and over time it will be averaged to almost zero [14]. The RPM was used with different cell types, and it could be shown that the altered gravity conditions favored the development of 3D-structures such as spheroids from thyroid cancer cells [15,16], duct-like constructs from breast cancer cells [17], 3D clusters from chondrocytes [18] and, most strikingly, intima-like tubular structures from endothelial cells [19]. Using the RPM might therefore allow a reduction in the number of cells, and the prefabrication of implants without the complications associated with scaffolds.

The objective of the present study was to demonstrate scaffold-free cartilage tissue formation from cryogenically preserved human chondrocytes. For this purpose, human articular chondrocytes were cultured for 7, 14, 21, and 28 days in two different media on the RPM running in the real random mode. After each time point, the spheroid production was investigated both macroscopically and microscopically to assess cell morphology and to determine the amount and distribution of cartilage-specific tissue components. Furthermore, quantitative real-time PCR (qPCR) analyses were conducted to investigate the expression of key genes involved in cartilage production.

## 2. Results

### 2.1. Chondrocyte Morphology Depends on the Cultivation Medium

**Figure 1:**
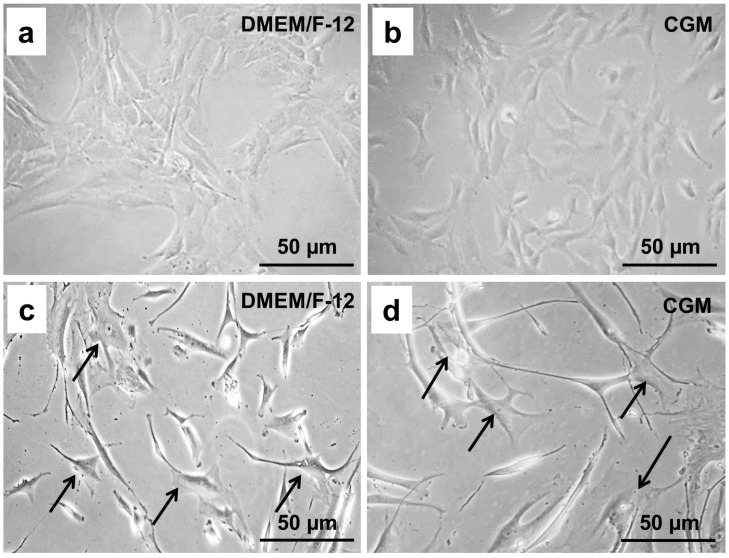
Morphological characteristics of human chondrocytes visualized by phase contrast microscopy. (**a**) Human chondrocytes cultivated in DMEM/F-12 medium after the first round of subculture. (**b**) Human chondrocytes cultivated in CGM after the first round of subculture. (**c**) Human chondrocytes cultivated in DMEM/F-12 medium after a third round of subculture. (**d**) Human chondrocytes cultivated in CGM after a third round of subculture. The arrows indicate the cell bodies of fibroblast-like single cells.

The cells responded differently to each medium tested. In the first passage, the chondrocytes cultivated in Dulbecco’s Modified Eagle Medium (DMEM)/F-12 medium and in chondrocyte growth medium (CGM), each supplemented with 10% fetal calf serum (FCS), displayed features of the chondrogenic phenotype; i.e., they were spherical, slowly proliferating, and formed colonies (Figure 1a,b).

Already after four rounds of subculture, the cells cultivated in both media showed abnormal morphology. They appeared dedifferentiated and formed a squamous eddy-like structure. We detected many rapidly growing fibroblast-like, star-shaped cells with a foamy, vacuolated cytoplasm (Figure 1c,d).

### 2.2. Chondrocytes Change Their Cellular Structure during Cultivation

**Figure 2:**
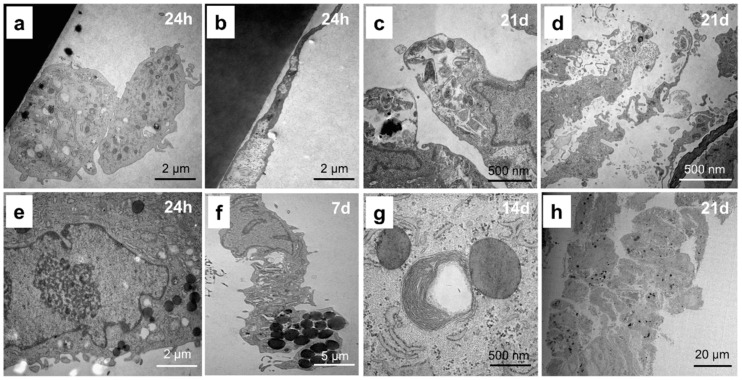
Morphological characteristics of chondrocytes analyzed by transmission electron microscopy. (**a**) Two chondrocytes cultivated for 24 h in DMEM/F-12 medium. (**b**) Partially edematous cytoplasm of chondrocytes cultivated for 24 h in DMEM/F-12 medium. (**c**) Chondrocytes undergoing autolysis during the cultivation for 21 d in DMEM/F-12 medium. (**d**) Cell edema after the cultivation for 21 d in DMEM/F-12 medium. (**e**) Cell lipids (black spots) visible after the cultivation for 24 h in DMEM/F-12 medium. (**f**) Cell lipids (black spots) visible after the cultivation for 7 d in DMEM/F-12 medium. (**g**) Cell lipids (black spots) visible after the cultivation for 14 d in DMEM/F-12 medium. (**h**) Cell lipids (black spots) visible after the cultivation for 21 d in DMEM/F-12 medium.

The chondrocytes are ovoid cells ranging in maximum diameter from about 10 µm in articular cartilage to about 30 µm in other hyaline cartilages [20] (Figure 2a). The cell has a scalloped surface (Figure 2a,b). The pericellular matrix is usually of a finer texture than the coarsely extracellular matrix more remote from the cell (Figure 2b). The junction is often sharply demarcated and constitutes the lacunar rim. The cell nucleus in older chondrocytes is often irregular (Figure 2c).

After endocytosis, the digestion of partly degraded macromolecules is completed in the lysosomal vacuole [21] with complete breakdown of the ECM around the cells (Figure 2c). Mitochondria are quite numerous in chondrocytes of immature tissue but in the adult become scarcer, smaller, and dense with few cristae (Figure 2c). This is in accord with the low respiratory activity of chondrocytes. However, in the growth plate, mitochondria are often the only organelles that are well-preserved. Moreover, prolonged cultivation resulted in cell edema, disturbing physiological processes, and doubling of the chondrocytes (Figure 2d).

Glycogens and lipids are common inclusions of the chondrocyte (Figure 2e–h). The large amounts of lipid in chondrocytes have never been explained satisfactorily. Fat is a normal inclusion, but there is little evidence that it is used either by the cell itself or by the whole organism. Fat globules increase in size during maturation and prolonged cultivation (Figure 2e–h) and are smaller in articular than other hyaline cartilages.

### 2.3. 24-h Short-Term Gene Expression Changes on the RPM

**Figure 3:**
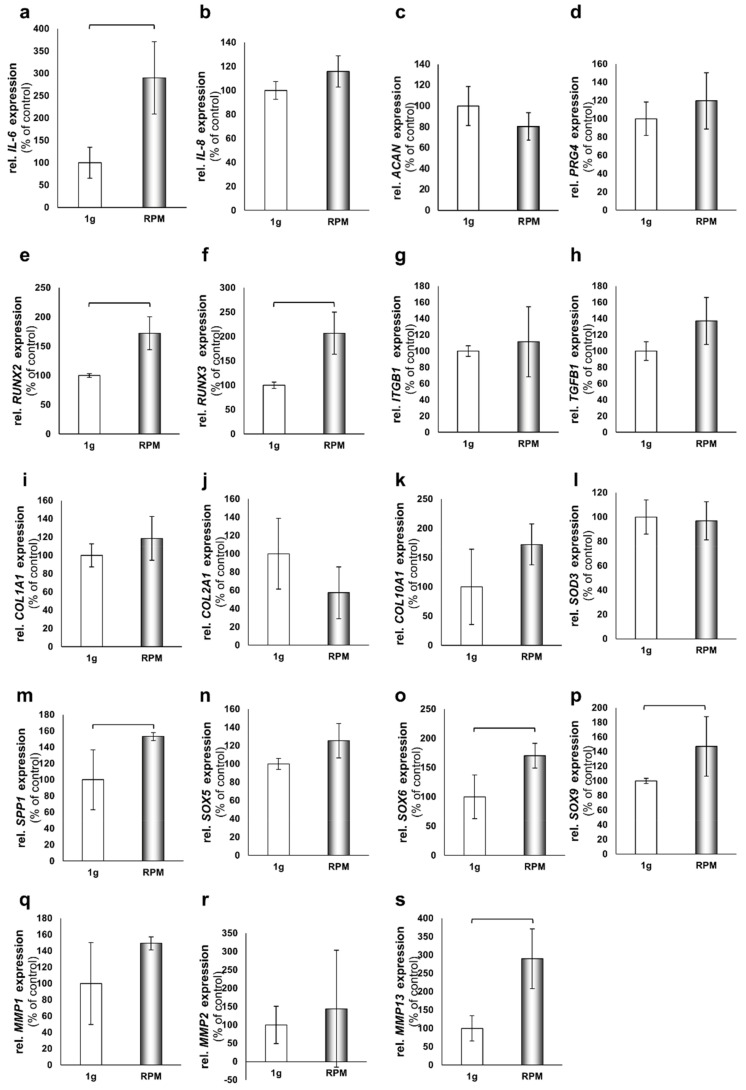
Quantitative real-time PCR analysis of cartilage-related genes. (**a**) IL6. (**b**) CXCL8 (IL8). (**c**) ACAN. (**d**) PRG4. (**e**) RUNX2. (**f**) RUNX3. (**g**) ITGB1. (**h**) TGFB1. (**i**) COL1A1. (**j**) COL2A1. (**k**) COL10A1. (**l**) SOD3. (**m**) SPP1. (**n**) SOX5. (**o**) SOX6. (**p**) SOX9. (**q**) MMP1. (**r**) MMP2. (**s**) MMP13. All analyses were done with chondrocytes cultivated in DMEM/F-12 medium for 24 h on the RPM. Static samples incubated beside the RPM inside the same incubator served as the 1 *g* controls. Brackets above the bars indicate significant changes (*n* = 5 each group: 1 *g* and RPM), significance *p* < 0.05).

To screen early adoption reactions of chondrocytes to the culture conditions on the RPM, we performed quantitative real-time PCR analyses after 24 h on chondrocytes cultured in DMEM/F-12. We focused on A: *IL6*, B: *IL8*, C: *ACAN*, D: *PRG4*, E: *RUNX2*, F: *RUNX3*, G: *ITGB1*, H: *TGFB1*, I: *COL1A1*, J: *COL2A1*, K: *COL10A1*, L: *SOD3*, M: *SPP1*, N: *SOX5*, O: *SOX6*, P: *SOX9*, Q: *MMP1*, R: *MMP2*, and S: *MMP13* (Figure 3). We found significant increases in the gene expressions of *IL6*, *RUNX2*, *RUNX3*, *SPP1*, *SOX6*, *SOX9*, and *MMP13*, while *IL8*, *ACAN*, *PRG4*, *ITGB1*, *TGFB1*, *COL1A1*, *COL2A1*, *COL10A1*, *SOD3*, *SOX5*, *MMP1*, and *MMP2* remained unchanged (Figure 3). No significant down-regulations were observed.

### 2.4. Chondrocytes Produce Spheroids on the RPM up to 2 mm in Size

**Figure 4:**
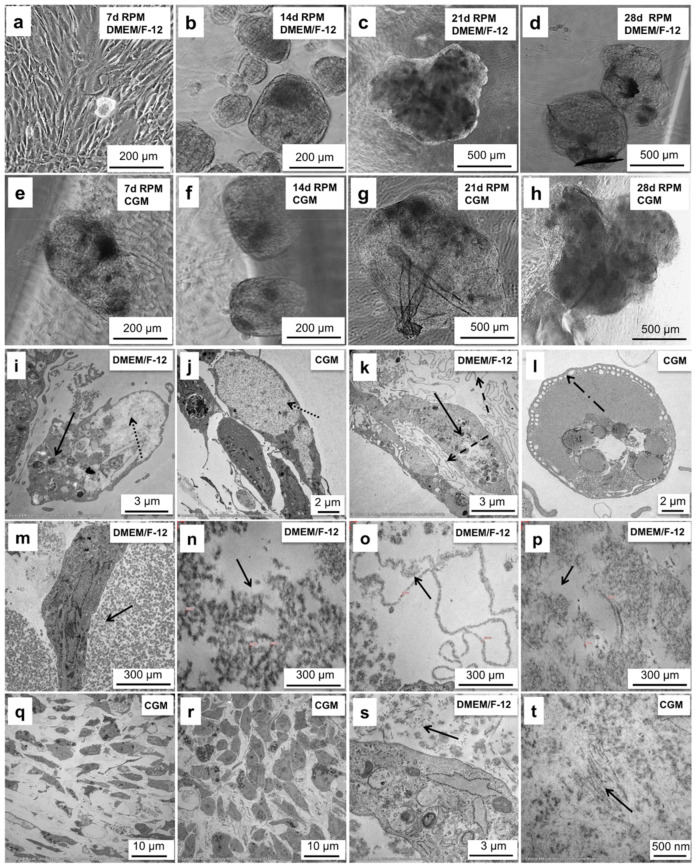
Morphological characterization of spheroids produced from articular chondrocytes on the RPM. (**a**) Spheroids produced during the 7-day-cultivation in DMEM/F-12 medium (**b**) Spheroids produced during the cultivation for 14 days in DMEM/F-12 medium. (**c**) Spheroids produced during the 21-day-cultivation in DMEM/F-12 medium. (**d**) Spheroids produced during the cultivation for 28 days in DMEM/F-12 medium. (**e**) Spheroids produced during the cultivation for 7 days in CGM medium. (**f**) Spheroids produced during the cultivation for 14 days in CGM medium. (**g**) Spheroids produced during the 21-day-cultivation in CGM medium. (**h**) Spheroids produced during the cultivation for 28 days in CGM medium. (**i**) Accumulation of glycogen (dotted arrow) and lysosomes (solid arrow) in the cells composing spheroid produced during the 28-day-cultivation in DMEM/F-12 medium. (**j**) Accumulation of glycogen (dotted arrow) in the cells composing spheroid produced during the 28-day-cultivation in CGM medium. (**k**) Cell edema (dashed arrow) and lipid (solid arrow) accumulation in the cells of the spheroid produced during the 28-day-cultivation in DMEM/F-12 medium. (**l**) Pinocytotic activity (dashed arrow) of the cells inside the spheroid produced during the 28-day-cultivation in CGM medium. (**m**) Granular clusters (arrow) of the material inside the spheroid produced during the 28-day-cultivation in DMEM/F-12 medium. (**n**) Variable long “zigzag“-forming filaments (arrow) inside the spheroid produced during the 28 d cultivation in DMEM/F-12 medium. (**o**) Loop organized structures (arrow) inside the spheroid produced during the 28 d cultivation in DMEM/F-12 medium. (**p**) Multifocal interspersed fine filament structures (arrow) inside the spheroid produced during the 28-day-cultivation in DMEM/F-12 medium. (**q**) Elongated cells on the surface of the spheroid formed during the 28-day-cultivation in CGM. (**r**) Round-shaped cells inside the spheroid formed during the 28-day-cultivation in CGM. (**s**) Collagen II bundles (arrow) inside the spheroid produced during the 28-day-cultivation in DMEM/F-12 medium. (**t**) Collagen II bundles (arrow) inside the spheroid produced during the 28-day-cultivation in CGM medium. Cells of passage 3 were used for the experiment.

After 7 d of sustained cultivation on the RPM in DMEM/F-12, we detected the formation of 3D spheroids (Figure 4a). Over the course of the experiment, the spheroids increased in size (Figure 4b,c) and reached about 1 mm in diameter after 28 d (Figure 4d).

Using CGM the spheroid production was detected already after 4 d of cultivation. After 7, 14, and 21 days they were bigger than their counterparts grown in DMEM/F12. (Figure 4e–g). On day 28, the spheroids reached about 2 mm in diameter (Figure 4h).

### 2.5. Chondrocyte Spheroids Have a Rich Morphology and Form Collagen Fibrils

After 28 d of cultivation in DMEM/F-12 medium, we observed an accumulation of lysosomes (Figure 4i), spherical vesicles containing hydrolytic enzymes, which are capable of breaking down proteins, nucleic acids, carbohydrates, lipids, and cellular debris inside the spheroids. Lysosomes function in the removal of effete intracellular material that are also concerned with the turnover of the extracellular matrix in chondrocytes [22].

Moreover, the cells accumulated glycogen (Figure 4i,j), a common polysaccharide inclusion of the chondrocyte that serves as main energy storage form of glucose in the body. Glycogen may be one of many sources of organic phosphate for calcification in the epiphyseal growth plate providing the raw material for matrix synthesis [22].

After 28 d of sustained cultivation of chondrocytes on the RPM, we detected cell edema (Figure 4k), abnormal accumulation of fluid, and fat accumulation (Figure 4k). Among these fats, cholesterol, for example, protects membrane integrity/cell viability and thus is able to change the shape of the membrane and move about.

In the chondrocytes cultivated in DMEM/F-12, we detected pinocytosis (Figure 4l), a mode of endocytosis used primarily for the absorption of extracellular fluids. Four different kinds of filaments have been detected in the conjunctive tissue of the spheroids (Figure 4m–p): (1) Granular clusters of the material looking like basement membranes (Figure 4m); (2) Variable long ”zigzag“ forming filaments (Figure 4n); (3) Loop-organized structures looking like basement membranes (Figure 4o); (4) Multifocal interspersed fine filament structures (Figure 4p). Most chondrocytes contain fine filaments 7–10 nm in diameter in the cytoplasm. Large masses of them are thought to be a sign of cell degeneration [23]. While actin-like filaments have been identified in cultured chondrocytes [24], the chemical nature of the 10 nm filaments is not known.

The cartilage-like tissue formed in CGM was composed of the cells of two different shapes—elongated (Figure 4q) and round (Figure 4r). It is generally accepted that chondrocyte morphology changes are dependent upon the location within articular cartilage. Cells near the surface are ellipsoid and become spheroidal with depth into the middle and deep zones [25].

After 28 d the cells formed proto-collagen fibrils, one of the most important components of the cartilage (Figure 4s,t). The fibrils are indeterminate in length and insoluble, and they form elaborate 3D arrays that extend over numerous cell lengths [26]. They have a distinctive 67 nm axial periodicity with a length in the range of several mm and range in diameter from a few to 500 nm (Figure 4t).

### 2.6. Cytoskeletal Composition of Spheroid Changes with the Duration of the Cultivation Time

**Figure 5:**
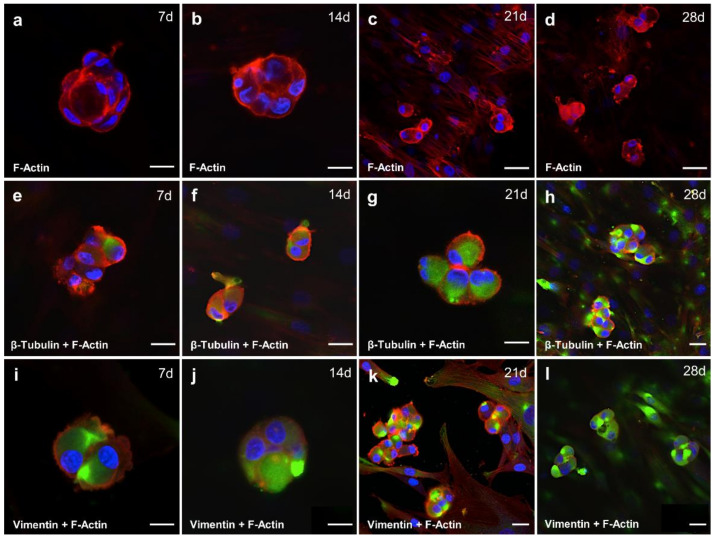
Cytoskeletal elements and arrangement in spheroids produced on the RPM. (**a**) F-Actin staining of spheroids produced during 7 d, (**b**) 14 d, (**c**) 21 d and (**d**) 28 d cultivation. (**e**) Immunofluorescence staining (IFS) of tubulin together with F-actin of spheroids produced during 7 d, (**f**) 14 d, (**g**) 21 d and (**h**) 28 d cultivation on the RPM. (**i**) IFS of vimentin together with F-actin of spheroids produced during 7 d, (**j**) 14 d, (**k**) 21 d, (**l**) and during 28-day-cultivation on the RPM. Scale bars = 20 µm; red (rhodamine-phalloidin): F-actin, blue: Hoechst dye 33342-stained nuclei, green (FITC): tubulin or vimentin.

It is now widely accepted that the cytoskeleton plays a role in sensing changes in gravity. It is composed of three types of protein networks: actin microfilaments, microtubules as represented by beta-tubulin staining, and intermediate filaments as represented by vimentin staining. Studies of the major components of the cytoskeleton in other cell types have shown that actin microfilaments play roles in a tremendous number of cellular events including migration [27], adhesion [28], shape alteration [29], cell signaling [30], and extracellular matrix assembly [31]. Under microgravity conditions, actin stress fibers are reduced in number, length, and thickness. Actin is often redistributed and has either a more perinuclear or more cortical localization [32], as shown by F-actin staining. The shape of the spheroids cultivated in DMEM/F-12 medium supplemented with 10% FCS was round and cortically localized stress fibers were visible on days 7, 14 and 21 of cultivation (Figure 5a–c,e–g,i–k) and still visible at day 28 with higher staining intensity (Figure 5d,h,l).

As constantly renewing microtubules contribute to intracytoplasmic transport [33], mitotic spindle formation [34], and ciliary movement [35], and they can be up-regulated in chondrocyte hypertrophy [36]. Under microgravity conditions, microtubules lose their radial organization, and they can be shortened, more curved, and bent [32]. They are regularly localized more perinuclearly in cells exposed to real and simulated microgravity; this has been detected during our experiments already after 7 d of cultivation on the RPM (Figure 5e). With the prolonged cultivation time, the staining intensity increased (Figure 5e–h).

Intermediate filaments were predominantly implicated in the mechanical integration of cellular space and in the spatial coordination of mechanical events [37] as well as signal transduction [38]. Intermediate filaments form clusters under simulated microgravity conditions as detected by the vimentin staining after 7 d (Figure 5i) and 14 d (Figure 5j). Moreover, larger meshes appear in the network, and the localization is more perinuclear [32], as detected on the 28th day (Figure 5l).

### 2.7. Cartilage-Like Tissue Reacts Positive Against Cartilage Markers and Produces Cartilage-Specific Proteins

**Figure 6:**
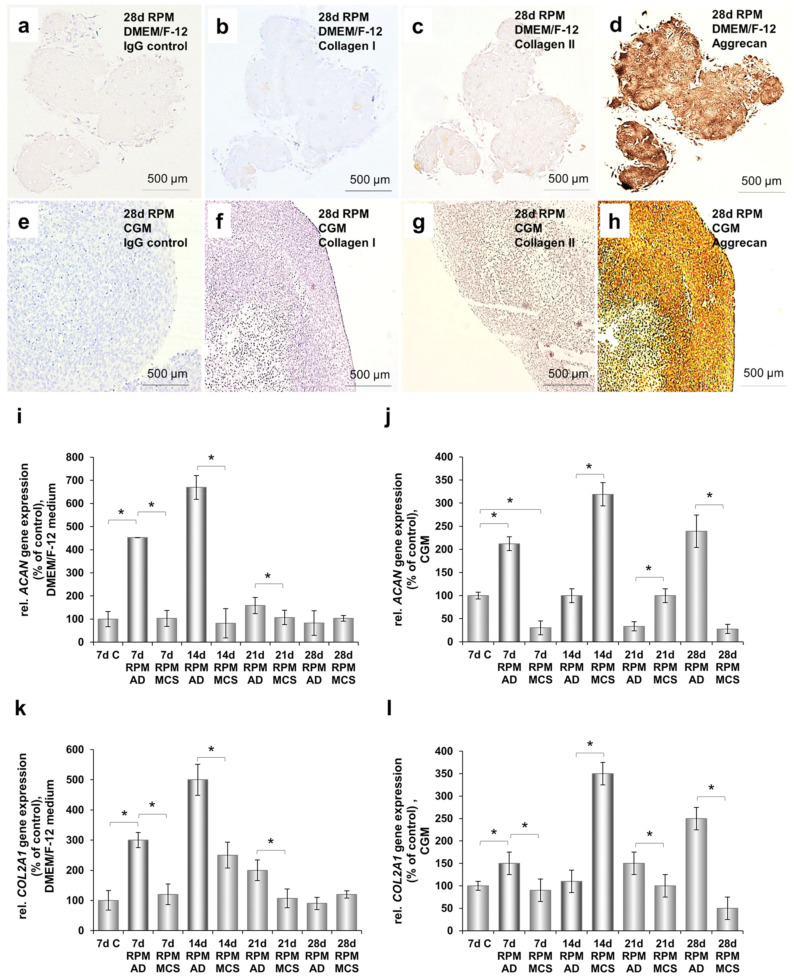
Identification and quantification of cartilage-specific components, using immunohistochemical staining and qPCR analyses. (**a**) Negative control of 28-day-old tissue produced in DMEM/F-12 medium stained only with the secondary antibody. (**b**) Immunohistochemical staining of 28-day-old spheroid produced in DMEM/F-12 medium with monoclonal antibody against collagen type I. (**c**) Monoclonal antibody against collagen type II, (**d**) Monoclonal antibody against aggrecan. (**e**) Negative control of 28-day-old tissue produced in CGM stained only with the secondary antibody. (**f**) Immunohistochemical staining of 28-day-old spheroid produced in CGM with monoclonal antibody against collagen type I, (**g**) with monoclonal antibody against collagen type II, and (**h**) with monoclonal antibody against aggrecan. (**i**) Relative gene expression of aggrecan in 7-day-old chondrocytes cultured under normal gravity conditions (C), and in adherent cells (AD) and tissue (MCS) produced in DMEM/F-12 medium after 7 d, 14 d, 21 d, and 28 d of RPM-exposure. (**j**) Relative gene expression of collagen type 2 in C, AD, and MCS produced in DMEM/F-12 medium after 7 d, 14 d, 21 d, and 28 d of RPM-exposure. (**k**) Relative gene expression of aggrecan in C; AD, and MCS produced in CGM after 7 d, 14 d, 21 d, and 28 d of RPM-exposure. (**l**) Relative gene expression of collagen type 2 in C; AD, and MCS produced in CGM after 7 d, 14 d, 21 d, and 28 d of cultivation on the RPM. C: 7-day-old chondrocytes cultured under normal gravity conditions; AD: adherent cells on the RPM; MCS: 3D tissue constructs on the RPM. All qPCR experiments were done with *n* = 5 replicates. * *p* < 0.05.

Morphological examination of sections of all produced tissue constructs from both cultivation media revealed differentiated chondrocytes ordered in clusters within a continuous dense cartilaginous matrix negative for collagen I (Figure 6b,f), but strongly positive for collagen type II (Figure 6c,g) and aggrecan immunostaining (Figure 6d,h). Moreover, in the spheroids produced in DMEM/F-12 with prolonged cultivation time, we detected increased gene expression of aggrecan (Figure 6i) and collagen II (Figure 6k) compared to the control cells cultivated for 7 d under normal gravity conditions.

Interestingly, in the spheroids produced in CGM we detected the highest aggrecan (Figure 6j) and collagen type II (Figure 6l) gene expression after 14 d of sustained cultivation.

### 2.8. Cartilage Tissue Production is Medium-Dependent

**Figure 7:**
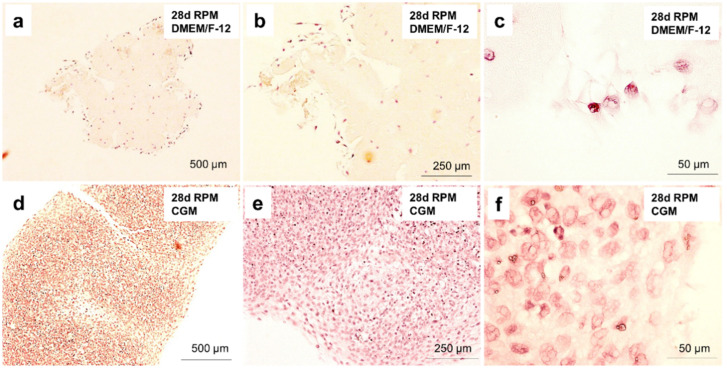
Histological analysis of cartilage-like tissue using Hematoxylin Eosin (HE) staining. (**a**) HE staining of spheroids produced on the RPM during the 28-day-cultivation in DMEM/F-12 medium. (**b**) Closer look at the cell content of the spheroids produced on the RPM during the 28-day-cultivation in DMEM/F-12 medium. (**c**) Closer look at the cell structure of the spheroids produced on the RPM during the 28-day-cultivation in DMEM/F-12 medium. (**d**) HE staining of spheroids produced on the RPM during the 28-day-cultivation in CGM. (**e**) Closer look at the cell content of the spheroids produced on the RPM during the 28-day-cultivation in CGM. (**f**) Closer look at the cell structure of the spheroids produced on the RPM during the 28-day-cultivation in CGM.

The cartilage produced on the RPM was analyzed employing histochemical methods (Figure 6). Using HE staining we detected a high amount of connective tissue with several isolated cells lying apart from each other (Figure 7a–c) in the spheroids grown in DMEM/F-12. This structural composition is typical for hyaline cartilage tissue. Hyaline cartilage subsequently appears as a very uniform, glossy type tissue with evenly dispersed chondrocytes in lacunae. Hematoxylin Eosin (HE) staining revealed that spheroids produced in CGM had a very cell-rich morphology (Figure 7d–f).

### 2.9. Spheroids Produced on the RPM Form Monolayers under 1 g Culture Conditions

**Figure 8:**
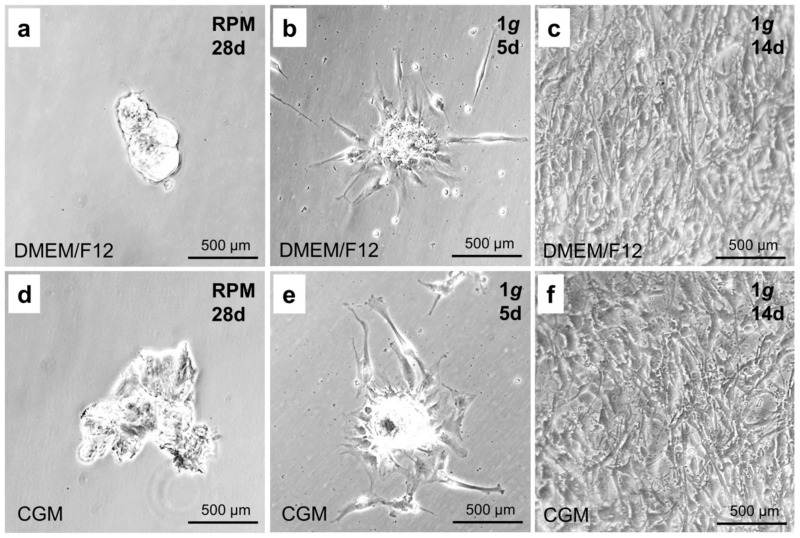
Spheroids produced during 28-day-cultivation on the RPM and re-cultivated under normal gravity conditions. (**a**) Microscopic analysis of spheroids produced after 28 days on the RPM in DMEM/F-12 medium. (**b**) Microscopic analysis of the spheroids produced after 28 days on the RPM in DMEM/F-12 medium after the cultivation for 5 days under normal gravity conditions (1 *g*). (**c**) Microscopic analysis of the spheroids produced after 28 days on the RPM in DMEM/F-12 medium after the cultivation for 14 days under normal gravity conditions (1 *g*). (**d**) Microscopic analysis of spheroids produced after 28 days on the RPM in CGM. (**e**) Microscopic analysis of the spheroids produced after 28 days on the RPM in CGM after the cultivation for 5 days under normal gravity conditions (1 *g*). (**f**) Microscopic analysis of the spheroids produced after 28 days on the RPM in CGM after the cultivation for 14 days under normal gravity conditions (1 *g*).

After transferring them into a fresh cell culture flask, the spheroids produced on the RPM after 28 d of sustained cultivation were floating on the surface of the cultivation medium for the first five days (Figure 8a,d). After that time, they sedimented and re-attached to the bottom of the cell culture flask. In addition, we detected several cells which began to spread out from the main cell mass (Figure 8b,e).

After 14 days, the cultivation flask was completely confluent (Figure 8c,f). These effects were independent of the type of culture medium and occurred at the same time and rate in both DMEM/F12 and CGM medium.

## 3. Discussion

Articular cartilage is the main load-bearing skeletal tissue of the synovial joint. It consists of an ECM made up of water (75%), collagen type II, proteoglycan, and chondrocytes originating from mesenchymal stem cells undergoing differentiation [38,39,40]. Despite the fact that chondrocytes sparsely contribute to the composition of adult articular cartilage, representing only about 1% of the cartilage volume [41,42], their declining activity during skeletal development [43] influences the formation, maturation, and aging of cartilage, which results in its degradation or in injuries which could lead to OA [44].

During spaceflights, astronauts experience a reduction of mechanical forces important for cartilage maintenance. This has a negative impact on load-bearing tissues including bone and skeletal muscle and may also affect cartilage integrity and homeostasis [45]. Single cells in vitro respond to changes in gravity, and this response might play an important role for physiological changes at the organism level during spaceflight [46]. As it is still under discussion how gravitational forces can exert their effects on the cellular level, altered gravity conditions (simulated or real) offer a unique environment to study the mechanical (gravity-dependent) response of cells [32].

It is well known that microgravity can be regarded as a tool to induce 3D-growth of human cells or even tissues changing cellular morphology, phenotype, and metabolic activity [47].

Tissue engineering of cartilage, i.e., the in vitro cultivation of cell-polymer constructs consisting of bovine articular chondrocytes on polyglycolic acid scaffolds, was investigated in an earlier study by Freed et al. [48]. Chondrocytes were grown first for 3 months on Earth and then for an additional 4 months on either MIR or on Earth in a bioreactor yielding cartilaginous constructs, each weighing between 0.3–0.4 *g* and consisting of viable, differentiated cells that synthesized proteoglycan and type II collagen [48]. Compared with the Earth group, MIR-grown constructs were more spherical and smaller.

**Figure 9:**
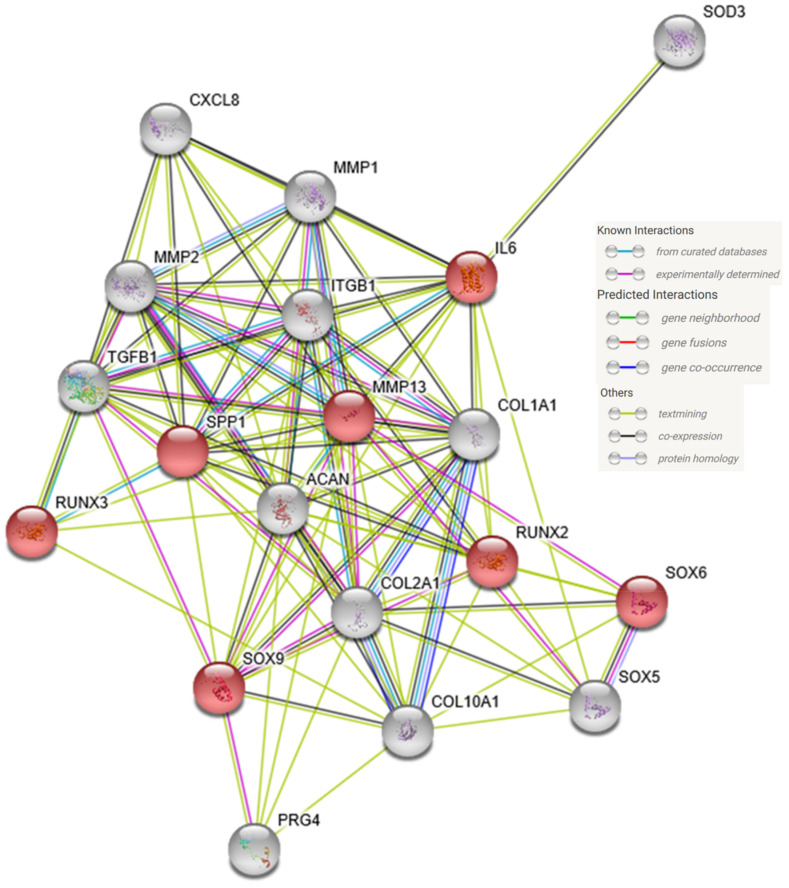
Mutual interaction network of the proteins encoded by the genes analyzed by quantitative real-time PCR. Colored lines represent different types of interaction as indicated in the legend. Red balls show up-regulated genes in qPCR, gray balls unregulated genes.

Interestingly, our qPCR studies after 24 h of exposure to the RPM indicate that the chondrocytes were under a certain degree of stress at this point and show in part increased gene expression of markers of OA and cartilage damage (Figure 9).

It has been shown that patients suffering from OA or rheumatoid arthritis had significantly higher concentrations of the pro-inflammatory cytokines IL6 and IL8 in their serum and synovial fluid than healthy subjects [49]. Furthermore, IL6 receptor blockade using tocilizumab preserved the articular cartilage in a mouse model of osteonecrosis [50]. Lastly, in accordance with our results, IL6 has been shown to stimulate MMP1, -3, and -13 via JAK-STAT and ERK-MAPK signaling in chondrocytes [51]. While the increase in *MMP1* gene expression in our experiment was not significant, *MMP13* expression was significantly increased by about 3-fold. Among the MMP family, MMP13 has the highest specificity for collagen type II and can also degrade aggrecan [52], thus promoting collagen degeneration. It is also almost exclusively expressed in chondrocytes, making MMP13 an interesting target for OA therapy [52].

Of note, osteopontin has also been found to be a regulator of MMP13 expression. While the majority of studies indicated that elevated osteopontin expression is associated with increased cartilage degeneration [53,54,55,56], suggesting that it is an inducer of MMP13 expression, there are some contrary findings [57]. However, our data supports the inducer-model, as we also found concomitantly increased *SPP1* and *MMP13* gene expression levels after 24 h on the RPM.

Beside those genes indicative of cartilage damage, we also observed the overexpression of genes involved in cartilage development, maintenance, and differentiation. The most central transcription factor is SOX9. It directly targets genes of important cartilage-specific extracellular matrix molecules (collagen type II, IX, XI aggrecan and link protein), their regulators such as chondroitin 4-sulfotransferase, related transcription factors such as SOX5 and -6 or RUNX2 and -3, and important cartilage signaling pathway mediators (fibroblast growth factor receptor-3) [58,59,60,61,62,63]. SOX9 and SOX5/6 constitute a trio which is sufficient for chondrogenesis. It binds to so-called super enhancers clustered throughout the genome to which the cartilage-specific genes are linked [62]. In addition, SOX9 shows non-transcriptional activities, most notably by interacting with RUNX2, acting as a repressor [64]. SOX9 knockdown caused apoptosis in round chondrocytes and terminal maturation with strong apoptosis in flat chondrocytes. It was therefore concluded that SOX9 expression is associated with chondrocyte survival [65].

On the other hand, RUNX2 and 3 are usually only weakly expressed in resting and proliferating chondrocytes but up-regulated in pre-hypertrophic, hypertrophic, and terminally hypertrophic chondrocytes, driving chondrocyte maturation and endochondral ossification [66,67,68]. In terminal hypertrophic chondrocytes, RUNX2 regulates the expression of *SPP1* and *MMP13*, among others [69,70,71,72]. This and the findings that *Runx2^+/−^* mice were resistant to OA [73] and that a *RUNX2* deletion resulted in an attenuation of the progression of OA in a surgically induced mouse model [74] indicate that RUNX2 plays a central role in the pathogenesis of OA.

Summarizing our qPCR findings, after 24 h on the RPM the chondrocytes were proliferating well, as indicated by their *SOX6/9* gene expression profiles. However, they also had undergone some microgravity-induced changes, which might be the first steps toward hypertrophy, matrix degeneration, and ossification. After a prolonged exposure to the RPM, however, these processes did not progress, but we found cartilage-like tissue constructs without signs of chondrocyte maturation or matrix depletion. We therefore propose that the chondrocytes, after the initial shock of simulated microgravity growth conditions, were able to adapt to cultivation on the RPM and suppress most of the deleterious processes.

The Rotating Wall Vessel (RWV) developed by NASA proved to be a useful tool for providing an environment that enabled dedifferentiated chondrocytes to re-differentiate and produce a cartilage-specific extracellular matrix. Dedifferentiated chondrocytes exposed to the RWV for 12 weeks also showed spontaneous aggregation and formation of solid tissue [75]. However, we had demonstrated earlier that human chondrocytes when exposed to the RPM start forming 3D cell assemblies within five days [18]. In this study, we also showed that initially after a 24-h RPM exposure, the cells revealed no signs of apoptosis [18]. The adherent cell layer on the RPM served as a starting point for the transition from monolayer cells to 3D spheroids which takes about 5–7 days [76]. Aleshcheva et al. reported the small 3D spheroids engineered on the RPM [76]. Moreover, the RPM-derived cartilage production has never been accelerated and the produced tissue has never been implemented into an animal model, which should be done in the future. According to our results, chondrocytes are able to produce cartilage tissue on the RPM already after 28 days of cultivation using DMEM/F-12 medium, demonstrating a strong positive staining with monoclonal antibodies against collagen type II and articular proteoglycan. Using chondrocyte-specific medium (CGM) it was possible to achieve comparable results after only 14 days.

Another approach for cartilage tissue engineering is the use of mesenchymal stem cells (MSC) derived either from bone marrow or from adipose tissue [77]. In general, simulated microgravity seemed to be beneficial in promoting the differentiation of MSC into a chondrogenic phenotype. Ohyabu and team demonstrated large tissues with a cartilaginous differentiation after exposing rabbit MSCs to an RWV for 4 weeks [78]. Similar results were reported by other authors, who demonstrated that growing MSCs under conditions of s-µg results in a better quality of the cartilage specimen in comparison to standard 1*g* spheroid culture techniques [79,80,81]. Furthermore, Yuge et al. showed that hMSCs exposed to 3D clinorotation were characterized by a strong proliferative potential and were able to differentiate into hyaline cartilage after transplantation [80]. It has to be mentioned that there are also controversial reports. Mayer-Wagner et al. used the RWV bioreactor and found a reduced chondrogenic potential of hMSCs during chondrogenic differentiation. Moreover, COL2A1 was likewise reduced and the COL2A1/COL10A1 ratio decreased under RWV conditions [82]. These data show that future studies are necessary to find the best method or device suitable for tissue engineering of cartilage. The RPM has proven to be suitable for tissue engineering purposes of various tissues such as vascular constructs and bone tissues [83,84,85]. Similar tubular constructs to those engineered on the RPM have been observed in space during the ESA-SPHEROIDS space mission [84,86].

In summary, a long-term RPM exposure of chondrocytes was suitable to engineer cartilaginous tissues expressing cartilage markers. With respect to cartilage regeneration approaches, our findings indicate that scaffold-free RPM-derived chondrocyte spheroids retain the ability to attach to surfaces and to spread out onto it, firmly anchoring the tissue construct to its new environment. We suggest that this characteristic will allow for an OA therapy in the future.

## 4. Materials and Methods

### 4.1. Cells and Culture Medium

Commercially available human chondrocytes (Provitro^®^, Berlin, Germany) were cultured in DMEM/F-12 medium (ThermoFisher Scientific, Waltham, Massachusetts, United States) and Chondrocyte Growth Medium (CGM; Provitro^®^, Berlin, Germany), both supplemented with 10% fetal calf serum (Biochrom^®^, Berlin, Germany) and antibiotics (100 IU penicillin/mL and 100 µg streptomycin/mL, Biochrom^®^, Berlin, Germany)

The cells from frozen stocks (passage 1) were grown in T175 cell culture flasks (175 cm^2^; Sarstedt, Nümbrecht, Germany) until sub-confluent layers (70–80%) were obtained. Afterwards, the cells were subcultured (passage 2) in 5 T175 cell culture flasks. After reaching 70% confluence, cells from 3 T175 (passage 3) were combined and 10^6^ cells were seeded into each of 30 T25 cell culture flasks (25 cm^2^; Sarstedt, Nümbrecht, Germany) for the RPM experiments (*n* = 15 for the RPM and *n* = 15 as 1 *g* control samples).

### 4.2. Random Positioning Machine

Altered gravity conditions were simulated using a desktop RPM, manufactured by Airbus Defense and Space Netherlands (formerly Dutch Space, Leiden, The Netherlands). The RPM is an instrument that can rotate a (biological) sample around all three axes in space, resulting in randomization of the influence of gravity. Whether this situation is experienced by the exposed systems such as “microgravity” has to be validated by space experiments and thus real microgravity conditions [87,88]. The RPM consists of two frames, which can be rotated independently, and are controlled by a dedicated software. To minimize residual acceleration, the samples were mounted as near to the center of rotation as possible and air bubbles avoided. Thirty T25 culture flasks containing confluent chondrocyte monolayers were completely filled with medium. Fifteen of these flasks were fixed on the RPM, which was then operated at an average angular velocity of 60°/s using the real random mode, varying both the speed and the movement direction of the frames. The RPM was positioned in a commercially available incubator at 37 °C and 5% CO_2_. Fifteen T25 culture flasks used for 1 *g* static ground control cultures were filled in parallel and placed in the same incubator as the RPM. Media were exchanged every 48 h.

After 7, 14, 21, and 28 days, three T25 flasks were removed from both the RPM and the control group, and adherent cells (RPM and controls) and spheroids (RPM) were collected for further investigations.

### 4.3. Histological Analysis

Following the RPM run, the spheroids were carefully removed from the medium after 7, 14, 21 and 28 d of cultivation, fixed in 4% paraformaldehyde (PFA; Sigma, Taufkirchen, Germany), embedded in paraffin, and sectioned into 3 µm thick slices. One slice from each group was stained with hematoxylin and eosin (Sigma, Taufkirchen, Germany) to gain an overview of the tissue structure and cell distribution.

### 4.4. Immunohistochemical Analyses

For immunohistochemistry, sections were deparaffinized according to standard procedures and stained with antibodies against human collagen type I, collagen type II, and aggrecan.

Briefly, slides were blocked for endogenous peroxidase activity with 2% hydrogen peroxide in DPBS for 15–20 min at room temperature. Then they were treated with pepsin (1 mg/mL; Sigma, Taufkirchen, Germany) in 0.5 M acetic acid for collagen types I and collagen II (30 min at 37 °C) and with chondroitinase ABC (0.2 U/mL; Boehringer Mannheim Biochemica, Mannheim, Germany) in 0.1 M Tris-acetate buffer at pH 8.0 for cartilage proteoglycan (2 h at 37 °C). The sections were rinsed with PBS and incubated for 30 min at 25 °C with normal goat serum diluted 1:10 in PBS. Primary antibodies were diluted in PBS–2% bovine serum albumin (BSA; Sigma) as follows: anti-collagen type I 1:100, anti-collagen type II 1:100, and anti-human aggrecan 1:50. The antibody solutions were then incubated with the sections in a humidified chamber for 1 h at room temperature. After incubation, the slides were washed three times with PBS and incubated for 1 h with biotin-conjugated goat anti-mouse secondary antibody (Vector Laboratories, Burlingame, CA, USA) diluted 1:100 in PBS/2% BSA. The slides were then treated with avidin–biotin (Vectastain ABC kit; Vector Laboratories) for 30 min and incubated with diaminobenzidine (DAB) substrate solution (Sigma) for approximately 10–15 min until the brown color developed. Then they were rinsed with PBS, counterstained with hematoxylin, rinsed with water, dehydrated, and finally sealed using Eukitt (Kindler, Freiburg, Germany).

All experiments included negative controls, which were treated with DPBS only, to test for unspecific binding by the secondary antibody.

### 4.5. Immunofluorescence Staining

For immunofluorescence staining, the cells (10^6^ cells/cm^2^) were seeded into several four-chamber Super Cell chamber slides (BD, Heidelberg, Germany) and placed in the incubator (37 °C, 5% CO_2_) overnight until they attached to the slides. The next day, the slides were completely filled with medium, sealed with parafilm, and placed on the RPM for the run. After the run, the chondrocytes were washed twice with DPBS, fixed for 30 min with 4% paraformaldehyde at 4 °C, and permeabilized with Triton X-100 (Sigma, Taufkirchen, Germany). The cells were then washed twice in DPBS and incubated with primary antibody for 24 h at room temperature. The morphology of the microtubules and intermediate filaments were determined by indirect immunofluorescence (IIF). After incubation with the primary antibody (beta-tubulin, 1:1000, vimentin, 1:1000; both Cell Signaling Technology, Inc., Danvers, MA, USA), the chondrocytes were washed twice with DPBS and incubated for 2 h with the secondary FITC-tagged antibody, used at a dilution of 1:500 (Cell Signaling Technology, Inc., Danvers, MA, USA). For nuclear staining, we used Hoechst dye 33342 (Molecular Probes, Eugene, OR, USA) for 5 min and washed the cells twice with DPBS. The cells were mounted with Vectashield^®^ immunofluorescence mounting medium (Vector, Burlingame, CA, USA), and analyzed microscopically.

### 4.6. F-Actin Staining

F-actin was visualized by means of rhodamine-phalloidin staining (Molecular Probes^®^, Eugene, OR, USA) [19,89]. For this, adherent cells were fixed for 30 min with 4% PFA (in DPBS), washed twice with DPBS, incubated with 5 µg/mL fluorescent phalloidin conjugate solution in PBS/1% BSA for at least 20 min at room temperature, and then washed several times with PBS to remove unbound phalloidin conjugate. Afterwards, the nuclei were stained with Hoechst 33342 (Molecular Probes^®^, Eugene, OR, USA) for 5 min and washed twice with DPBS. For evaluation, the samples were mounted with Vectashield^®^ (Vector, Burlingame, CA, USA) and analyzed microscopically.

### 4.7. Microscopy

The viability and morphology of the cells grown on four-chamber Super Cell slides (BD, Heidelberg, Germany) were examined by phase contrast microscopy (Olympus, Hamburg, Germany) immediately after RPM exposure. Immunofluorescence and F-actin staining were analyzed with a Zeiss 510 META inverted confocal laser scanning microscope (Zeiss, Germany) equipped with a Plan-Apochromat 63 × 1.4 objective. Excitation and emission wavelengths were as follows: λ_exc_ = 488 nm and λ_em_ = 505 nm for FITC. All samples were analyzed with the help of the image analysis program Scion Image (Version 1.63 MacOs, Scion Corporation, Chicago, IL, USA).

### 4.8. Transmission Electron Microscopy (TEM)

Following the RPM run, the spheroids derived from passage 3 chondrocytes were fished from the medium after 7 d, 14 d, 21 d and 28 d cultivation and fixed in Karnovsky fixative. The samples were then embedded in EMbed-812 resin blocks using a LYNX EI tissue processor (Leica Biosystems, Nussloch, Germany). After a first assessment of semi-thin sections (0.8 µm, toluidine blue/fuchsin double staining) by light microscopy, representative blocks were chosen for further ultra-thin sectioning (80 nm). Contrasting was done with Pb-citrate and U-acetate. TEM analyses were performed with a LEO912AB (Zeiss, Oberkochen, Germany) instrument, equipped with a 2k × 2k pixel side-entry CCD-camera (TRS, Moorenweis, Germany) and the iTEM image analysis software (OSIS, Münster, Germany).

### 4.9. RNA Isolation

Ten cell culture flasks from each time point were used for RNA extraction. The cells were scraped off using cell scrapers (Sarstedt, Nümbrecht, Germany), transferred to 50 mL tubes, and pelleted by centrifugation (2500 *g*, 10 min, 4 °C). The RNeasy Mini Kit (Qiagen, Hilden, Germany) was used according to the manufacturer’s instructions to isolate total RNA. RNA concentrations and quality were determined spectrophotometrically at 260 nm using a NanoDrop instrument (Thermo Scientific, Wilmington, DE, USA). The isolated RNA had an A260/280 ratio of 1.5 or higher. cDNA designated for quantitative real-time PCR was then obtained using the First-Strand cDNA Synthesis Kit (Fermentas, St. Leon-Rot, Germany) using 1 µg of total RNA in a 20-µL reaction mixture at room temperature.

### 4.10. Quantitative Real-Time PCR

Quantitative real-time PCR [90,91,92] was used to determine the expression levels of selected genes after 24 h or 7 d, 14 d, 21 d, and 28 d incubation under simulated microgravity (µg) compared to the static control group (1 *g*). Primer Express^®^ software (Applied Biosystems, Darmstadt, Germany) was employed to design all primers with a T_m_ of about 60 °C. The primers were synthesized by TIB Molbiol (Berlin, Germany) and are given in Table 1. All assays were run on an Applied Biosystems 7500 Fast Real-Time PCR System using the Fast SYBR^®^ Green PCR Master Mix (both Applied Biosystems, Darmstadt, Germany). The reaction volume was 25 µL including 1 µL of template cDNA and a final random hexamer primer concentration of 500 nM. PCR conditions were as follows: 20 s at 95 °C, 40 cycles of 3 s at 95 °C, and 30 s at 60 °C, followed by a melting curve analysis step (temperature gradient from 60 to 95 °C with +0.3 °C/cycle).

If all amplicons showed a single T_m_ similar to the one predicted by the Primer Express^®^ software; the PCR reactions were considered specific. Every sample was measured in triplicate. The comparative C_T_ (ΔΔC_T_) method was used for the relative quantification of transcription levels. 18S rRNA was used as a housekeeping transcript to normalize expression data.

### 4.11. STRING-Analysis

To generate the interaction network of genes analyzed by qPCR, we used the STRING V11.0 tool [93] (available at https://string-db.org/).

### 4.12. Statistical Analysis

All statistical analyses were performed using IBM SPSS Statistics 24.0 (IBM, Armonk, NY, USA). We tested all parameters achieved by qPCR analyses using one-way ANOVA or the Mann-Whitney U test (depending on the results of a normality test). All data are expressed as means ± standard deviation (SD). Differences were considered significant at *p* < 0.05.

## 5. Conclusions

Exposure of human chondrocytes to the RPM and thus a new environment by random positioning increased the current knowledge about tissue engineering of cartilage. We could demonstrate that the cultivation medium has a direct impact on the speed of 3D formation and tissue composition. The 3D tissues revealed cartilaginous characteristics that make them potential candidates for cellular cartilage regeneration approaches in OA therapy. Moreover, the results initiated by gravitational biomedical research provide a new technology to support the development of patient-specific therapies and a challenge for future applications in translational regenerative medicine.

## Figures and Tables

**Table 1:** List of primers used for qPCR analyses.

Gene	Forward Primer	Reverse Primer
*18S rRNA*	GGAGCCTGCGGCTTAATTT	CAACTAAGAACGGCCATGCA
*ACAN*	AGTCCAACTCTTCAAGGTGAACTA	ACTCAGCGAGTTGTCATGGT
*COL1A1*	ACGAAGACATCCCACCAATCAC	CGTTGTCGCAGACGCAGAT
*COL2A1*	GGCAATAGCAGGTTCACGTACA	CGATAACAGTCTTGCCCCACTT
*COL10A1*	GGGCAGAGGAAGCTTCAGAAA	TCTCAGATGGATTCTGCGTGC
*IL6*	CGGGAACGAAAGAGAAGCTCTA	GAGCAGCCCCAGGGAGAA
*CXCL8*	TGGCAGCCTTCCTGATTTCT	GGGTGGAAAGGTTTGGAGTATG
*ITGB1*	GAAAACAGCGCATATCTGGAAATT	CAGCCAATCAGTGATCCACAA
*MMP1*	GTCAGGGGAGATCATCGGG	GAGCATCCCCTCCAATACCTG
*MMP2*	CCATGATGGAGAGGCAGACA	CCATGATGGAGAGGCAGACA
*MMP13*	AGCCTTCAAAGTTTGGTCCGA	TCGCCATGCTCCTTAATTCCA
*PRG4*	CCCCCAAACCACCAGTTGTA	ACGTGTCAGGAGTTGTGACC
*RUNX2*	GAACCCAGAAGGCACAGACA	GGATGAGGAATGCGCCCTAA
*RUNX3*	GTGGGCGAGGGAAGAGTTTC	CCTTGATGGCTCGGTGGTAG
*SOD3*	CTGGAAAGGTGCCCGACTCC	ATGTCTCGGATCCACTCCGC
*SOX5*	TCCTCCCTCCAGGCTTCAG	CTGCCATGGTAGTTGGGATCA
*SOX6*	GCCACACATTAAGCG	TCCAGCGAGATCCTAAGATTTTG
*SOX9*	AGGAAGTCGGTGAAGAACGG	CGCCTTGAAGATGGCGTTG
*SPP1*	CGAGGTGATAGTGTGGTTTATGGA	CGTCTGTAGCATCAGGGTACTG
*TGFB1*	CACCCGCGTGCTAATGGT	AGAGCAACACGGGTTCAGGTA

All sequences are given in 5′-3′ direction.

## Abbreviations

µ*g*	Microgravity
ACAN	Aggrecan
bFGF	Basic fibroblast growth factor
CGM	Chondrocyte growth medium
COL10A1	Collagen alpha-1(X) chain
COL1A1	Collagen, type I, alpha 1,
COL2A1	Collagen, type II, alpha 1
DMEM	Dulbecco’s Modified Eagle Medium
ECM	Extracellular matrix
EGF	Epidermal growth factor
ERK	Extracellular signal-regulated kinase
FCS	Fetal calf serum
IL6	Interleukin 6
IL8	Interleukin 8
ITGB1	Integrin beta-1
JAK	Janus kinase
MAPK	Mitogen-activated protein kinase
MMP1	Matrix metalloproteinase-1
MMP2	Matrix metalloproteinase-2
MMP13	Matrix metalloproteinase-13
PFA	Paraformaldehyde
OA	Ostheoarthritis
PRG4	Proteoglycan 4
qPCR	Quantitative real-time PCR
R3 IGF-1	R3 insulin-like growth factor-1
RPM	Random Positioning Machine
RUNX2	Runt-related transcription factor 2
RUNX3	Runt-related transcription factor 3
RWV	Rotating Wall Vessel
SOD3	Extracellular superoxide dismutase
SOX5	SRY-related HMG-box transcription factor 5
SOX6	SRY-related HMG-box transcription factor 6
SOX9	SRY-related HMG-box transcription factor 9
SPP1	Osteopontin
STAT	Signal transducer and activator of transcription
TEM	Transmission electron microscopy
TGFB1	Transforming growth factor beta 1
VEGF	Vascular endothelial growth factor
